# Unilateral Vogt-Koyanagi-Harada Disease With Two Distinct Choroidal Neovascular Membranes: A Case Report

**DOI:** 10.7759/cureus.66405

**Published:** 2024-08-07

**Authors:** Raeba Mathew, Boopathy Murugavel

**Affiliations:** 1 Ophthalmology, Canadian Specialist Hospital, Dubai, ARE; 2 Ophthalmology, Aster Hospital, Al Qusais, Dubai, ARE

**Keywords:** secondary cnv, reactivation of vkh disease, peripapillary cnv, inflammatory choroidal neovascular membrane (cnv), unilateral vogt-koyanagi-harada (vkh) disease

## Abstract

Vogt-Koyanagi-Harada (VKH) disease is a multisystem inflammatory disease that usually presents with bilateral panuveitis. Unilateral manifestations of VKH, albeit rare, have been described. Choroidal neovascularization may occur as a complication during the disease. Reactivation of uveitis may also occur during treatment.

A patient with unilateral features of VKH disease presented with two distinct types of choroidal neovascular membranes and two episodes of reactivation of posterior uveitis. He underwent treatment with a combination of systemic steroids/immunosuppressive agents, intravitreal anti-vascular endothelial growth factor (anti-VEGF) agents, and dexamethasone implants with good clinical response.

Patients with VKH disease need to be diagnosed early, treated adequately with a combination of systemic and ocular medication, and followed up diligently for any complication that may arise, to optimize visual acuity.

## Introduction

Vogt-Koyanagi-Harada (VKH) disease is a multisystem, inflammatory, autoimmune disorder affecting melanocyte-containing tissues in the eye, inner ear, meninges, and integumentary system [1]. Ocular manifestations are usually bilateral, presenting as diffuse granulomatous panuveitis, predominantly choroiditis. Four phases have been described: (1) prodromal phase, with neurologic and auditory symptoms such as headache, vertigo, fever, meningismus, scalp hypersensitivity, and orbital pain, (2) acute uveitic phase, with bilateral diffuse choroiditis, multifocal exudative retinal detachment, optic disc hyperemia, and edema with or without anterior uveitis, (3) convalescent phase, with chronic granulomatous anterior uveitis, sunset glow fundus, integumentary changes such as vitiligo, poliosis, and alopecia, and auditory manifestations such as dysacusis, tinnitus, or hearing loss, and (4) chronic recurrent phase, with recurrent, chronic iridocyclitis. Most ocular complications such as cataract (42%), glaucoma (27%), choroidal neovascularization (CNV) (11%), and subretinal fibrosis (6%) manifest in the last two stages [2].

VKH is usually bilateral but may be asymmetrical, as in the original reports by Harada and Koyanagi [1,2]. True unilateral presentation of VKH is extremely rare and has been contested [3]. CNV may develop in VKH as a response to chronic inflammation or secondary to damage of the retinal pigment epithelium (RPE)-Bruch's membrane complex or a combination of both mechanisms [4]. In VKH, CNV are usually macular, mostly extrafoveal, rarely subfoveal, or associated with a chorioretinal scar. Peripapillary CNV in VKH is very rare, although cases have been reported [5,6].

We would like to report a unique case that presented with unilateral VKH and two rare, distinct types of CNV at different time points during the disease.

## Case presentation

A 28-year-old Indian male presented with a history of blurred vision in his right eye for one week. He was diagnosed with unilateral VKH six months previously elsewhere and treated with three doses of intravenous methylprednisolone 1 g, followed by oral prednisolone 60 mg tapered slowly, with improvement of vision. He had been on 5 mg once daily for two weeks when he noticed blurred vision again in the right eye. There was no history of prodromal symptoms prior to his initial visual symptoms, previous ocular trauma, or surgery. All laboratory investigations and brain MRI were within normal limits.

**Figure 1:**
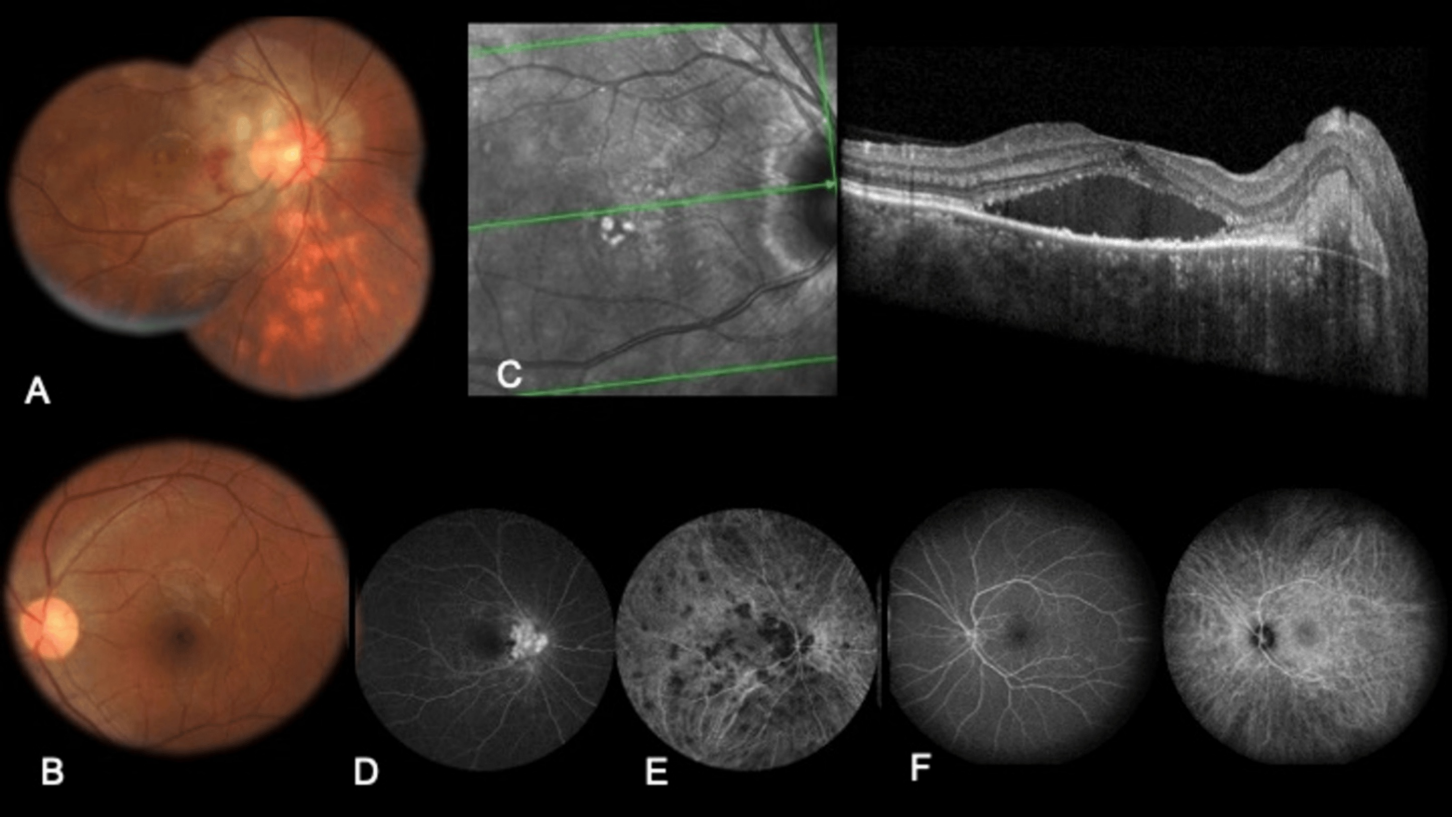
Baseline picture Color fundus picture of the right eye (A) shows optic disc edema with patches of intraretinal hemorrhage over the nasal macula and adjacent macular edema. The inferior retina shows a sunset glow appearance. Color fundus picture of the left eye (B) appears normal. OCT of the right eye (C) shows intraretinal thickening, subretinal fluid, subretinal hyperreflectivity in the peripapillary area, and increased choroidal thickness. FFA of the right eye (D) shows early peripapillary hyperfluorescence with late leak depicted here at 10 minutes. Multiple areas of hypofluorescent dark spots are seen in the posterior pole and outside the arcades on ICGA (E) in the right eye, the mid-phase picture at 10 minutes shown here. The left eye (F) is within normal limits on FFA and ICGA OCT: optical coherence tomography; FFA: fundus fluorescein angiography; ICGA: indocyanine green angiography

On examination, the best corrected visual acuity in the right eye was 6/18 and the left eye was 6/6. The anterior segment was quiet in both eyes. The right eye showed optic disc edema with patches of intraretinal hemorrhage over the nasal macula with no evidence of vitritis. The inferior retina showed a sunset glow appearance. Optical coherence tomography (OCT) showed intraretinal thickening, subretinal fluid (SRF) involving the fovea, subretinal hyperreflectivity in the peripapillary area, and increased choroidal thickness. Fundus fluorescein angiography (FFA) showed early peripapillary hyperfluorescence with late leak. Multiple areas of hypofluorescent dark spots were seen in the posterior pole and outside the arcades on indocyanine green angiography (ICGA) appearing early and persisting in the late images, in the right eye. The left eye was within normal limits clinically and on imaging (Figure 1).

A diagnosis of unilateral VKH disease with peripapillary choroidal neovascular membrane was made. The patient received an intravitreal injection of aflibercept. The dose of oral prednisolone was increased to 60 mg (1 mg/kg body weight), and the patient noted improvement in vision of the right eye with resolution of optic disc edema and macular SRF. 

**Figure 2:**
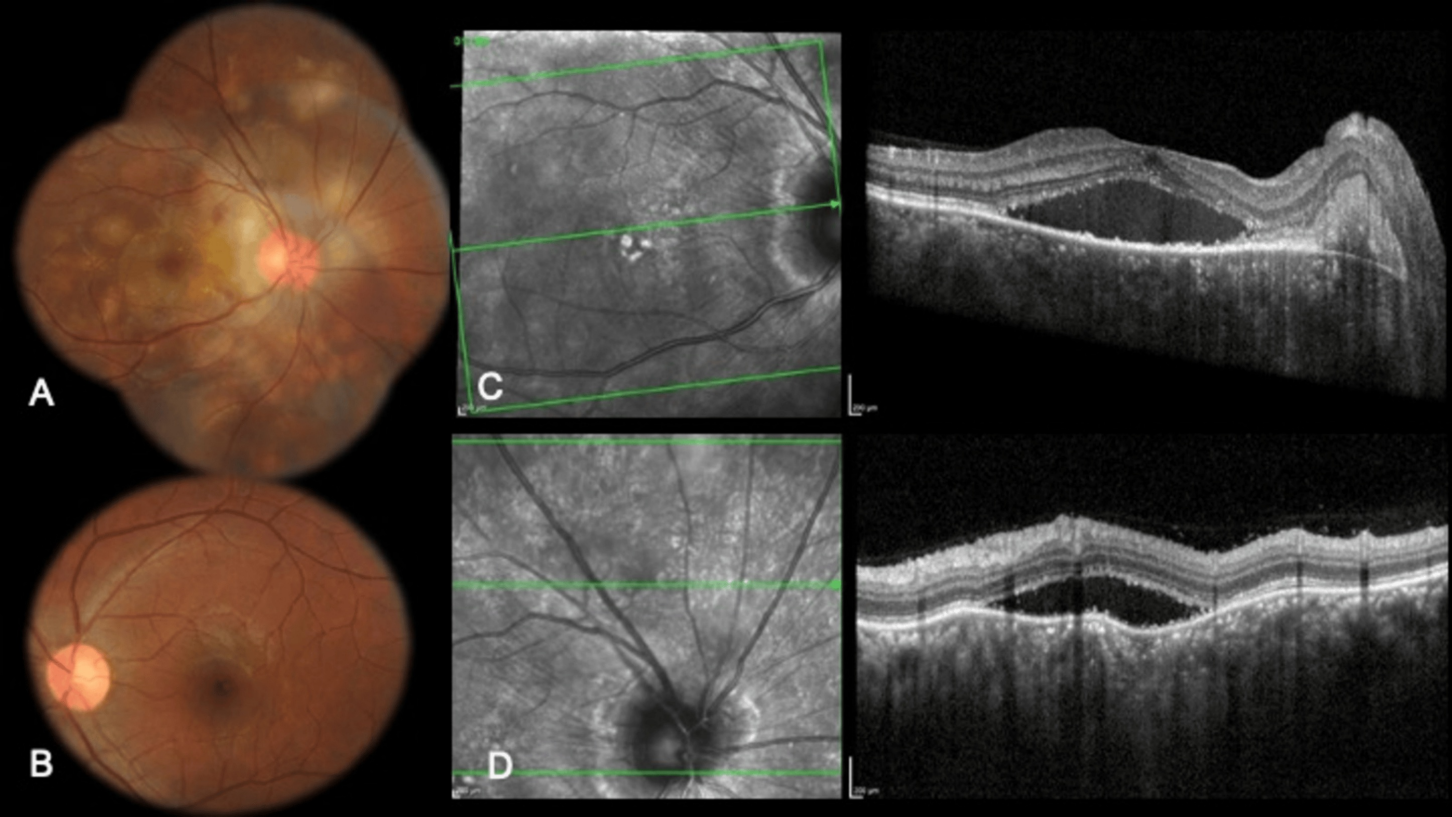
Reactivation of VKH Color fundus picture of the right eye (A) shows optic disc edema, multiple pockets of SRF over the posterior pole and outside the arcades extending to the mid-peripheral retina, and macular edema. Color fundus picture of the left eye (B) appears normal. OCT of the right eye (C) shows foveal SRF. OCT through the superior retina (D) shows SRF with underlying RPE undulations and increased choroidal thickness VKH: Vogt-Koyanagi-Harada; SRF: subretinal fluid; OCT: optical coherence tomography; RPE: retinal pigment epithelium

However, on tapering the oral dose of steroids to 40 milligrams after a month, the patient developed worsening vision in the right eye that showed increased optic disc edema, multiple pockets of SRF over the posterior pole and outside the arcades extending to the mid-peripheral retina, and macular edema. These findings were confirmed on OCT that also showed underlying RPE undulations, foveal SRF, and increased choroidal thickness (Figure 2). 

This was diagnosed as reactivation of posterior uveitis in the right eye. The left eye was within normal limits clinically and on imaging. The dose of prednisolone was increased to 1 mg/kg body weight, and the patient was commenced on oral methotrexate 15 mg once weekly and adalimumab 40 mg. Vision improved and there was subsidence of SRF and optic disc edema. The peripapillary CNV showed scarring with no evidence of leakage on OCT. 

**Figure 3:**
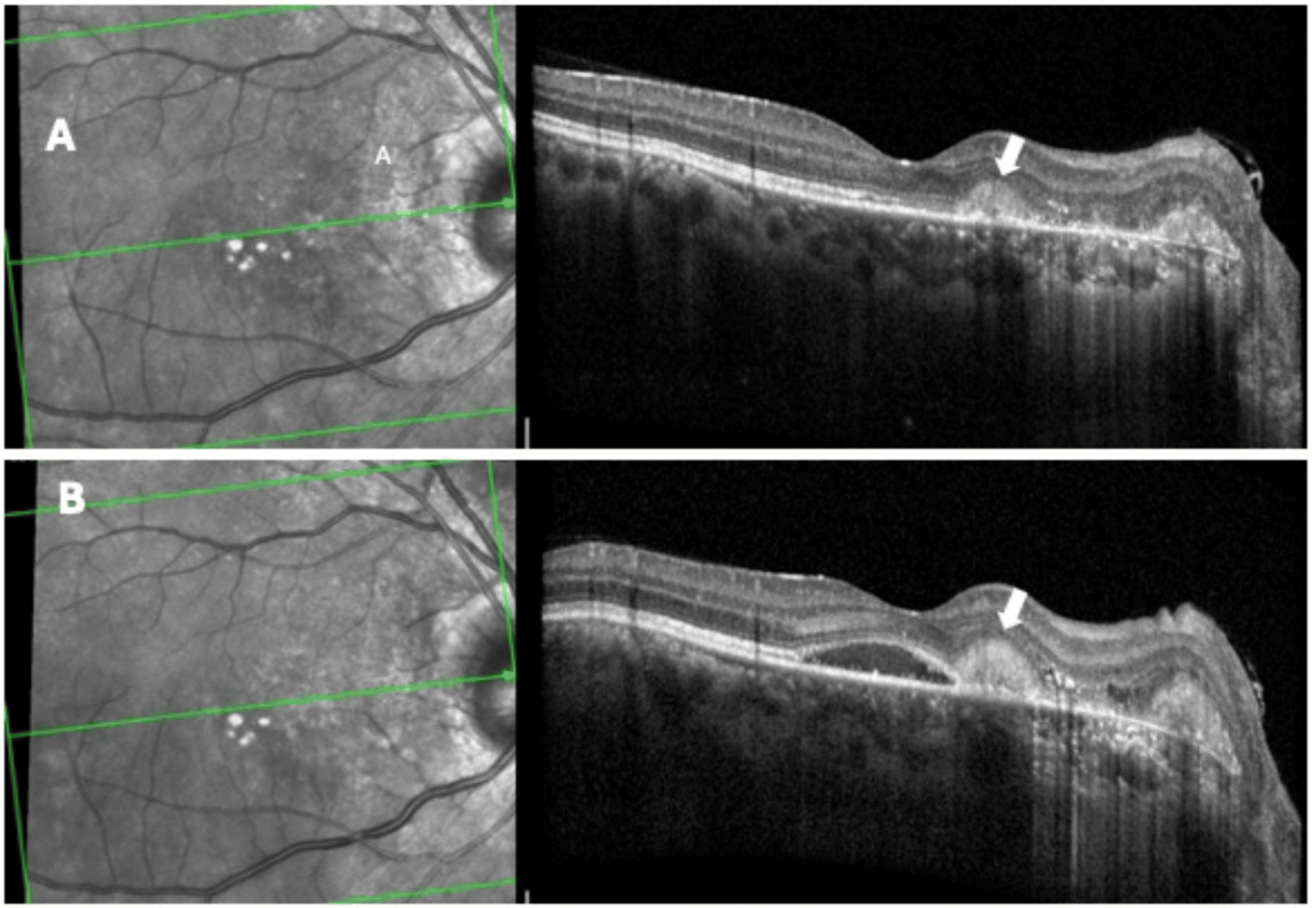
Macular neovascularization A small area of new hyperreflectivity (arrow) is noted on OCT of the right eye (A) in the papillomacular bundle area away from the optic disc. OCT of the right eye (B) shows subretinal hyperreflectivity in the nasal parafoveal area (arrow) with increased intraretinal thickening and cysts and subfoveal SRF, indicating active macular CNV OCT: optical coherence tomography; SRF: subretinal fluid; CNV: choroidal neovascularization

On follow-up after five months, the patient had completed four doses of adalimumab, oral prednisolone tapered to 5 mg daily, and oral methotrexate 10 mg weekly. The visual acuity improved to 6/6p in the right eye and the left eye remained at 6/6. OCT showed a dry macula, subfoveal ellipsoid zone disruption with preserved external limiting membrane, and peripapillary scar. However, a small area of new hyperreflectivity (arrow) was noted on OCT in the papillomacular bundle area away from the optic disc (Figure 3A).

Three months later, while the patient was on oral prednisolone 5 mg and oral methotrexate 10 mg weekly, he reported blurring of vision in the right eye. OCT showed an increase of subretinal hyperreflectivity in the nasal parafoveal area with intraretinal cysts and subfoveal SRF, indicating active macular neovascularization (arrow) with no evidence of posterior uveitis (Figure 3B). The left eye was within normal limits.

The patient underwent two intravitreal injections of aflibercept in the right eye a month apart, and a good response was noted clinically and on OCT. His vision improved to 6/7.5 in a month.

**Figure 4:**
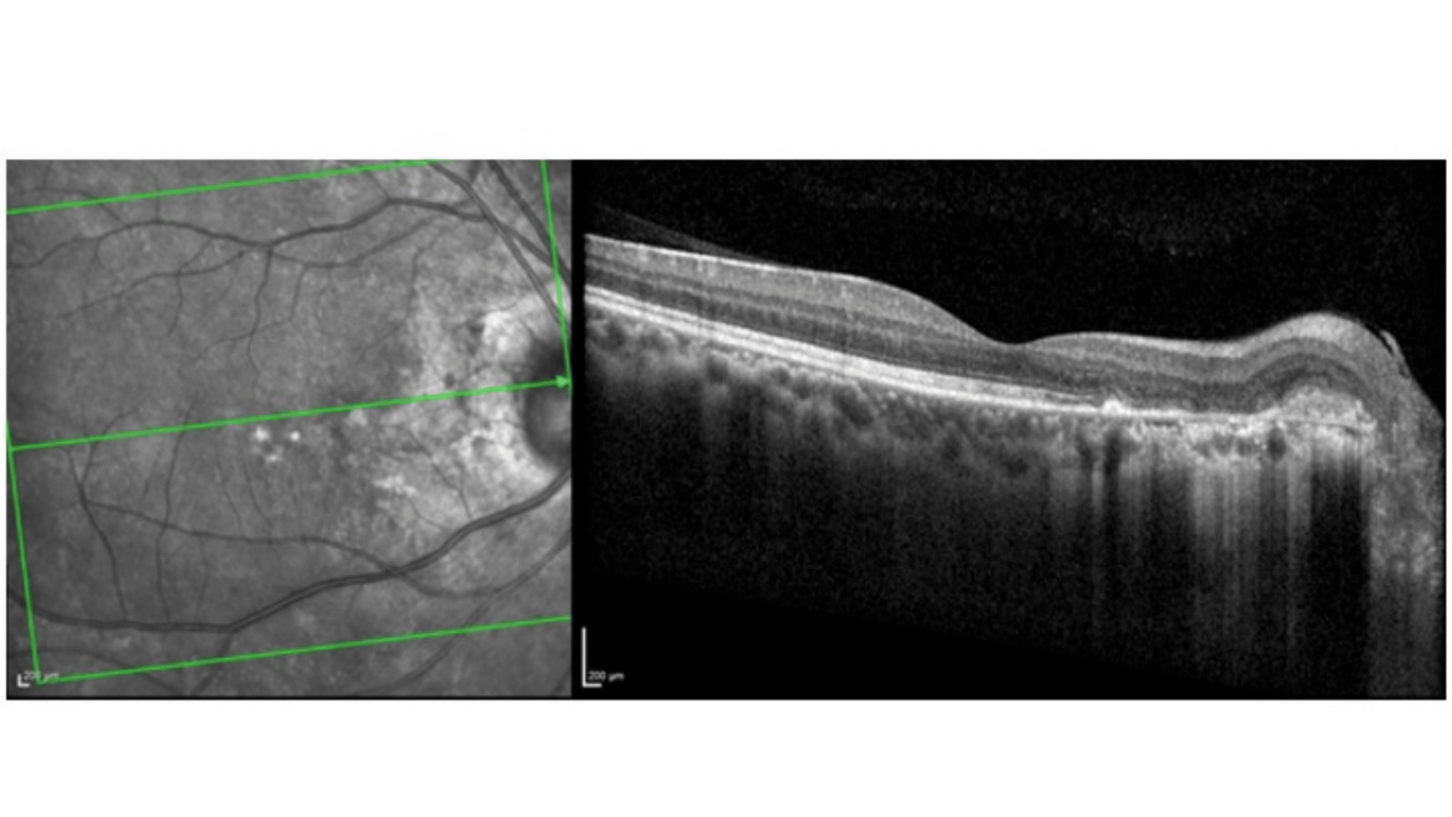
Final outcome At the last visit, the right eye retina appears stable, with reconstitution of outer retinal layers in the subfoveal area and visual acuity of 6/6

However, two months later, the patient developed vitritis and a flare-up of VKH in the right eye and received an intravitreal implant of dexamethasone 700 µg following which the posterior uveitis settled. He was tapered off oral steroids after two months and oral methotrexate after six months. On the last follow-up, 30 months from baseline, visual acuity is 6/6 in both eyes, the patient remains symptom-free, and the retina appears stable, with reconstitution of outer retinal layers in the subfoveal area (Figure 4).

## Discussion

Ocular VKH disease is considered a primary stromal choroiditis. It is usually bilateral but may be asymmetrical, with the other eye having subclinical involvement and less prominent inflammation, as in the original reports by Harada and Koyanagi [1,2]. Although several cases of unilateral VKH have been reported in recent decades, it has been suggested that subclinical inflammation in the other eye was undetected due to the lack of evaluation of the choroid [3]. Early use of immunosuppressive treatment with corticosteroids may also prevent the manifestation of subclinical inflammation in asymmetrical cases [3].

The mainstay of treatment in VKH is the initial aggressive suppression of intraocular inflammation by systemic corticosteroids, commenced as loading doses of either intravenous methylprednisolone or oral prednisolone [7]. However, systemic side effects of corticosteroids and the anticipated long duration of treatment in VKH necessitate concurrent systemic immunosuppressive therapy with tapering of the dose of steroids and complete stoppage of the former. The latter includes antimetabolites (methotrexate, azathioprine, and mycophenolate mofetil), alkylating agents (chlorambucil and cyclophosphamide), or antibiotics (cyclosporine and tacrolimus). Biologic drugs such as anti-tumor necrosis factor (anti-TNF) medications (adalimumab and infliximab) and anti-vascular endothelial growth factor (anti-VEGF) agents have recently been added to the armamentarium of drugs. Although these drugs are better tolerated than corticosteroids, vigilant monitoring for side effects needs to be done [8].

CNV may develop in VKH either due to angiogenic drive mediated by chronic inflammation or secondary to damage of RPE-Bruch's membrane complex or a combination of both mechanisms [4]. In VKH, CNV are usually macular and mostly extrafoveal; however, they may also be located in the subfoveal area or associated with a chorioretinal scar. They have been described as type 2 lesions that present with intraretinal fluid or SRF, hemorrhage, and exudation [9]. The natural history and prognosis of inflammatory CNV in VKH are more favorable than those in age-related macular degeneration (AMD), probably due to the younger age at presentation, smaller size, and type of membrane [10]. Peripapillary CNV in VKH is very rare, although cases have been reported [5,6].

Management of inflammatory CNV requires a combined approach to address the angiogenic drive with systemic immunosuppressive agents along with intravitreal anti-VEGF agents [11]. Prior to the advent of anti-VEGF agents, systemic corticosteroids were the mainstay of treatment for inflammatory CNV. This helped in the stabilization of the CNV in 80% of the cases [8,12]. Anti-VEGF agents are currently advocated as the first-line medication for the management of inflammatory CNV. Various reports of the use of bevacizumab, ranibizumab, and aflibercept in VKH-associated inflammatory CNV have shown a favorable response [6,13-15]. Most of the eyes responded well to the initial injection followed by variable time for reactivation necessitating pro re nata (PRN) treatment. A definite anti-VEGF protocol does not apply in the case of inflammatory CNV. An initial dose followed by PRN doses was seen to provide good anatomical and visual outcomes [12].

Subtenon injections of triamcinolone acetonide or intravitreal dexamethasone implant alone or in conjunction with systemic steroids have been reported to be effective in controlling inflammation in VKH disease [16,17].

We would like to highlight the rare occurrence of unilateral presentation of VKH in our patient. The initial peripapillary CNV is also an unusual finding in VKH. This patient had a protracted course with the appearance of two distinct CNV in the same eye. By the time the patient initially presented to us, a sunset glow was already present in the right eye, indicating the chronic stage of the disease. Clinical examination as well as retinal and choroidal imaging revealed persistent evidence of underlying VKH with active peripapillary CNV in the right eye. The left eye did not show any features of underlying VKH attributable to the early use of steroids that may have suppressed manifestation in that eye [3]. The initial peripapillary CNV was likely inflammatory; increasing the dose of systemic steroids along with an intravitreal injection of aflibercept helped in the control of the same. The second macular CNV after 10 months was found along an area of outer retinal disruption, separate from the initial CNV, with no evidence of inflammation in the eye at the time; hence, it may be considered secondary to damage of RPE-Bruch's membrane complex. This responded well to two injections of aflibercept, and the patient remained symptom-free for two months, until the reactivation of uveitis. Although the disease involved the peripapillary and nasal macular area, he still had preserved retinal integrity in the sub- and parafoveal regions, with normal visual acuity at the last follow-up.

The patient had two episodes of flare-up of posterior uveitis, the initial one possibly due to a sub-optimal immunosuppressive regime that allowed the persistence of inflammatory cytokines such as TNF-alpha, which was subsequently addressed with adalimumab and systemic methotrexate. The second episode was controlled by an intravitreal dexamethasone implant that helped to contain the inflammation. There were no further flare-ups for 30 months.

## Conclusions

Early diagnosis of VKH and prompt, adequate treatment are vital for controlling the disease and preventing permanent sight-threatening complications. Regular, diligent follow-up is essential not only for assessing the control of the ocular disease but also for monitoring any complications that may arise during the disease. A combination of ocular and systemic treatments helps maintain optimal visual acuity.

Our patient underwent treatment for a total duration of two years after he was initially seen in the clinic. Initial tapering of immunosuppression led to reactivation; hence, slow tapering of the same and the addition of adalimumab helped in the control and resolution of inflammation. Accurate diagnosis of etiology of the CNV helped in the appropriate management of the same.
